# Status quo of ALK testing in lung cancer: results of an EQA scheme based on in-situ hybridization, immunohistochemistry, and RNA/DNA sequencing

**DOI:** 10.1007/s00428-021-03106-5

**Published:** 2021-06-25

**Authors:** Philipp Jurmeister, Claudia Vollbrecht, Korinna Jöhrens, Daniela Aust, Anke Behnke, Albrecht Stenzinger, Roland Penzel, Volker Endris, Peter Schirmacher, Annette Fisseler-Eckhoff, Jens Neumann, Thomas Kirchner, Reinhard Büttner, Sabine Merkelbach-Bruse, Hans Kreipe, Danny Jonigk, Wolfram Jochum, Regulo Rodriguez, Manfred Dietel, David Horst, Michael Hummel, Maximilian von Laffert

**Affiliations:** 1 Institute of Pathology, Charité-Universitätsmedizin Berlin, Corporate Member of Freie Universität Berlin, Humboldt-Universität Zu Berlin and Berlin Institute of Health, 10117 Berlin, Germany; 2 German Cancer Consortium (DKTK), Partner Site Berlin, and German Cancer Research Center (DKFZ), 69210 Heidelberg, Germany; 3 German Cancer Research Center (DKFZ), Im Neuenheimer Feld 280, 69120 Heidelberg, Germany; 4 Institute of Pathology, University Hospital Dresden, 01307 Dresden, Germany; 5 Quality Assurance Initiative for Pathology (QuIP - Quality in Pathology) GmbH, Berlin, Germany; 6 Institute of Pathology, University Hospital Heidelberg, 69120 Heidelberg, Germany; 7 German Cancer Consortium (DKTK), Partner Site Heidelberg, 69120 Heidelberg, Germany; 8 Institute of Pathology, Helios Dr. Horst-Schmidt-Kliniken, 65199 Wiesbaden, Germany; 9 Department of Pathology, Faculty of Medicine, LMU Munich, 80337 Munich, Germany; 10 German Cancer Consortium (DKTK), Partner Site Munich, 80337 Munich, Germany; 11 Institute of Pathology, Faculty of Medicine and University Hospital of Cologne, 50937 Cologne, Germany; 12 Medical School Hannover, Institute of Pathology, 30559 Hannover, Germany; 13 Institute of Pathology, Cantonal Hospital St, 9007 GallenGallen, Switzerland

**Keywords:** Non-small cell lung cancer, Anaplastic lymphoma kinase, External quality assessment, Round robin, Next-generation sequencing

## Abstract

With this external quality assessment (EQA) scheme, we aim to investigate the diagnostic performance of the currently available methods for the detection of ALK alterations in non-small cell lung cancer on a national scale, namely, in situ hybridization (ISH), immunohistochemistry (IHC), and RNA/DNA sequencing (NGS). The EQA scheme cohort consisted of ten specimens, including four ALK positive and six ALK negative samples, which were thoroughly pretested using IHC, ISH, and RNA/DNA NGS. Unstained tumor sections were provided to the 57 participants, and the results were retrieved via an online questionnaire. ISH was used by 29, IHC by 38, and RNA/DNA sequencing by 19 participants. Twenty-eight institutions (97%) passed the ring trial using ISH, 33 (87%) by using IHC, and 18 (95%) by using NGS. The highest sensitivity and interrater agreement (Fleiss ‘ kappa) was observed for RNA/DNA sequencing (99%, 0.975), followed by ISH (94%, 0.898) and IHC (92%, 0.888). However, the proportion of samples that were not evaluable due to bad tissue quality was also higher for RNA/DNA sequencing (4%) compared with ISH (0.7%) and IHC (0.5%). While all three methods produced reliable results between the different institutions, the highest sensitivity and concordance were observed for RNA/DNA sequencing. These findings encourage the broad implementation of this method in routine diagnostic, although the application might be limited by technical capacity, economical restrictions, and tissue quality of formalin-fixed samples.

**Supplementary Information:**

The online version contains supplementary material available at 10.1007/s00428-021-03106-5.

## Introduction

Alterations of the anaplastic lymphoma kinase (ALK), most commonly in form of a paracentric inversion resulting in an EML4-ALK fusion transcript, occur in about 4–6% of non-small cell lung cancer (NSCLC) [1, 2]. Patients harboring this alteration can benefit from therapy with various tyrosine kinase inhibitors (TKI). Fluorescence in situ hybridization (FISH) has been used in the clinical trials that led to the approval of the first ALK TKI Crizotinib and is still considered the gold standard [3, 4]. However, numerous studies also demonstrated the reliability of immunohistochemistry (IHC) to identify patients with ALK alterations [5–11]. Furthermore, IHC is widely deployed and requires less technical expertise compared with FISH, making it a highly promising screening tool [12]. Beyond IHC and in situ hybridization (ISH), next generation-sequencing (NGS) panels are able to identify the distinct ALK-fusion transcripts, which seems of interest, as recent data showed that specific ALK-subtypes (e.g., EML4-ALK variant 3) may be clinically more aggressive and tend to show an earlier resistance to therapy [13]. Furthermore, nowadays, diagnostic cancer approaches need to cover far more than one alteration. This fact is of emerging importance as the number of genes of interest for targeted therapy keeps growing, while the amount of tissue available for investigation is often limited [14, 15]. Therefore, methods that can reliably identify multiple, predictive, or prognostic alterations within a single analysis are becoming increasingly important. Additionally, DNA/RNA sequencing methods have been shown to be very useful in cases where IHC or ISH give contradictory or inconclusive results [16, 17].

While several ALK ring trials have demonstrated a high interrater concordance between different institutions with regard to IHC and ISH, there is virtually no experience for RNA/DNA sequencing-based methods [6, 18, 19]. To address this, the Qualitätssicherungs-Initiative Pathologie GmbH (QuIP, Quality Assurance Initiative Pathology) initiated a ring trial, investigating the reliability of the three methods to correctly assess the ALK status of pretested NSCLC samples in a multicentric setting.

## Materials and methods

### Case selection

A total of ten cases were selected for the ring trial, including cases from the archives of the Institutes of Pathology of the Charité–University Hospital Berlin, the Heidelberg University Hospital, the University Hospital Cologne, the Ludwig Maximilian University of Munich, and the Medical School Hannover. The selection included four ALK positive and six ALK negative specimens. All cases had been pretested thoroughly by the institute that provided the samples and were retested centrally at the Charité–University Hospital Berlin, yielding concordant results for IHC, ISH, and RNA/DNA sequencing for all ten tumor samples.

### Construction of test sets and quality control (internal ring trial)

**Fig. 1:**
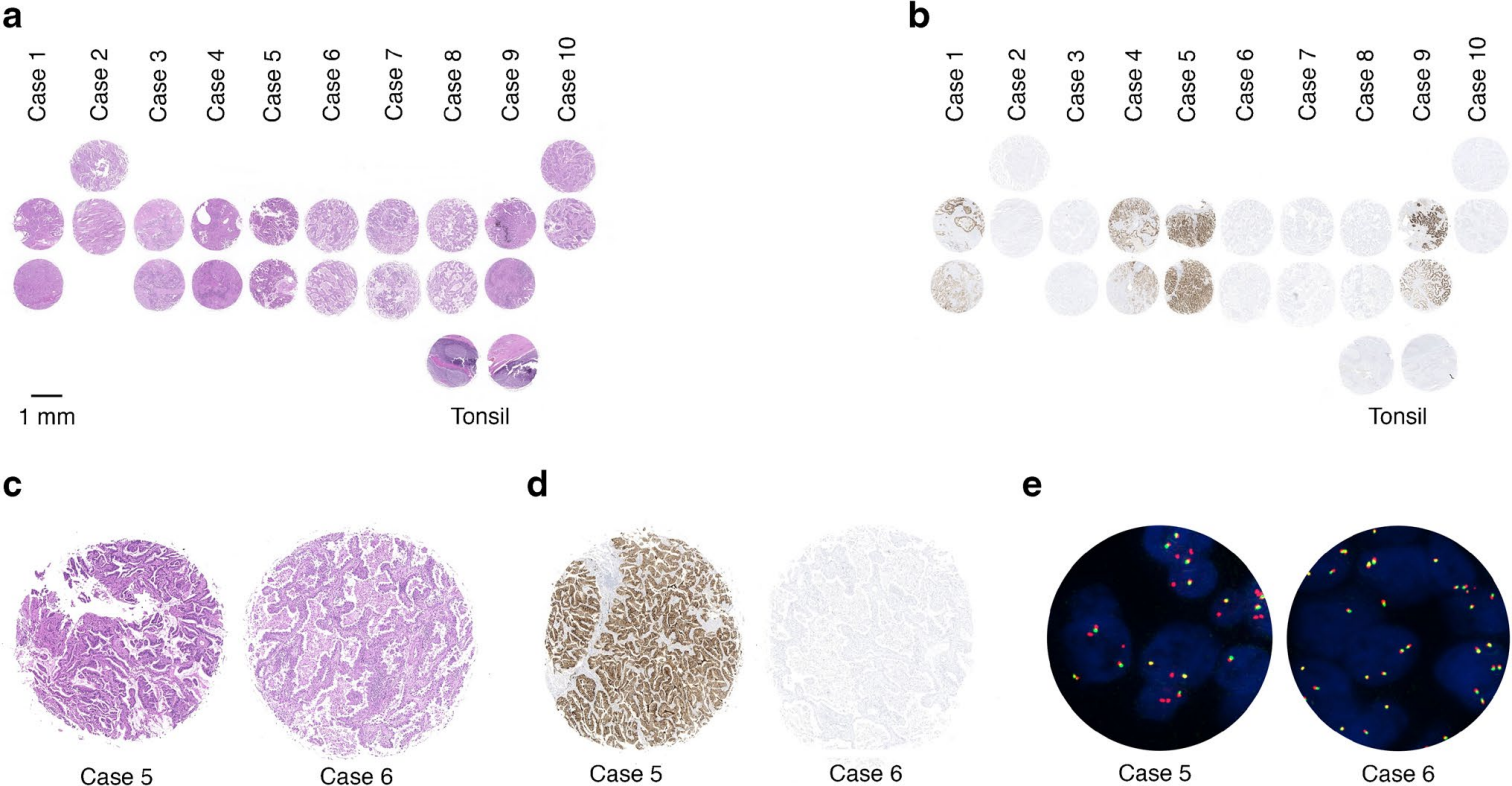
Tissue microarray (TMA) design and exemplary pictures for positive and negative results from ALK immunohistochemistry (IHC) and fluorescence in-situ hybridization (FISH)**. a** TMA design including two 1.5-mm cores for each of the ten selected cases, arranged in ten columns. For orientation and control purposes, two landmarks consisting of normal tissue from the palatine tonsil are located in the bottom right corner. **b** Overview of the results from ALK IHC: strong immunoreactivity can be observed in four samples, while six cases remained negative. **c** Hematoxylin and eosin stained sections of cases 5 and 6. **d** ALK IHC slides of cases 5 and 6. **e** Fish of cases 5 and 6: split signals and single red signals can be observed for case 5, indicating an ALK translocation/inversion. There is no indication for an ALK rearrangement in case 6

For IHC and ISH, two tissue microarrays (TMA) were constructed (Multiblock, Hannover, Germany) using the ten pretested specimens. For each case, two representative cores with a diameter of 1.5 mm were arranged in ten columns (Fig. 1a). For orientation and control purposes, two points of reference consisting of normal tissue from the palatine tonsil were also included in the TMA. Sections with a thickness of 2 µm (IHC) or 4 µm (ISH) were cut and two consecutive, unstained slides were provided to the participants. For RNA/DNA sequencing, representative tumor areas covering an area of at least 5 × 5 mm with a tumor cell content of at least 70% were macrodissected and embedded in separate paraffin blocks. For each participant, three consecutive sections per case with a thickness of 10 µm were provided.

To ensure that the selected tumor areas were representative and that the results were still in line with the previous ALK testings, both TMAs and the ten individual paraffin blocks for RNA/DNA sequencing were re-evaluated by seven expert institutes as part of a pretesting (“internal ring trial”). The sections for each method were re-evaluated by two different institutions and by the Institute of Pathology of the Charité–University Hospital Berlin. The investigators were blinded to the results from pretesting. Concordant results were achieved, so the samples were considered as suitable for the external quality assessment (EQA scheme).

Following the successful re-evaluation, the sections for the actual EQA scheme test were cut. The last sections of the test sets for all three methods were again tested at the Institute of Pathology at the Charité—University Hospital Berlin. The results for IHC and NGS were still in line with the previous testings. However, regarding the paraffin block for ISH, the tumor material from Case 5 had been used up during the production of the test sets. With the help of H&E stained slides, it was possible to determine that for this case only normal tissue was included in the last eight test sets. These sets were still sent out to the participants, however, “negative” or “not evaluable, tissue not representative” was expected as correct answers.

### Execution of the EQA scheme and certification

The EQA scheme was rolled out in Germany and Switzerland. Upon registration, all participants were asked to select their method or methods of choice, as all techniques required different material types. All participants were free to enroll for one or up to three techniques.

All slides were cut, stored at 4 °C and sent to the participants within 16 days. Representative H&E slides were digitalized and provided to the institutions via online access. All participants had three weeks to complete the analyses and to submit the results via an online questionnaire.

All cases had to be classified as “positive,” “negative,” “not evaluable, tissue not representative,” or “not evaluable, technical issues.” A correct result was rewarded with two points, whereas no point was given for an incorrect evaluation. If a case was classified as “not evaluable,” one point was given, but only accepted for one case. Thus, the maximum score was 20 points. In line with general EQA scheme evaluation policy by the QuIP, at least 18 points (90%) were required for successful participation. The results had to be returned within 21 days.

The questionnaire included additional, non-mandatory questions, covering technical details on the respective method and the institute’s routine diagnostic approach to ALK testing. However, these answers were not required for successful participation.

### Statistical analysis

Statistical analysis was performed using RStudio version 1.1.463 based on the statistical language R version 3.5.1 [20, 21]. The *irr* package was used to calculate the Fleiss’ kappa value for multiple raters [22]. The Fleiss’ kappa values were interpreted as followed: as 0 = poor agreement, 0.010–0.200 = slight agreement, 0.210–0.400 = fair agreement, 0.410–0.600 = moderate agreement, 0.610–0.800 = substantial agreement, and 0.810–1 = almost perfect agreement [23].

## Results

### Participants

**Fig. 2:**
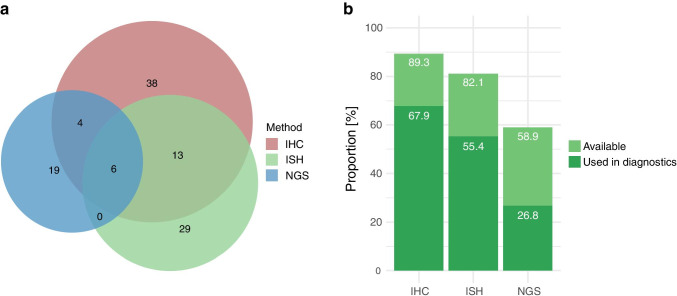
Distribution of methods used in the ring trial as well as in routine diagnostics**. a** Venn diagram showing the distribution of the selected methods for the ALK ring trial between the participants. 38, 29 and 19 institutions applied for immunohistochemistry (IHC), in-situ hybridization (ISH) and next-generation sequencing (NGS), respectively. Thirteen participants used IHC and ISH, four used IHC and NGS and six used all three methods. **b** Stacked bar plots summarizing the methods that were generally available for ALK testing at the participating institutions and the actual use of these techniques in the diagnostic setting

Overall, 57 institutions registered for the ring trial, including 56 participants from Germany and one participant from Switzerland. As all centers were free to apply for one or more methods, there were a total of 86 registrations, including 38 for IHC, 29 for ISH, and 19 for RNA/DNA sequencing. Thirty-four (60%) participants enrolled for one, 17 (30%) for two and six (11%) for all three methods (Fig. 2a).

The participants were also asked about the availability of the three methods for ALK testing and which of those techniques were actually used in routine diagnostic (independently from the use of those methods in the ring trial). Data on these questions was available for 56 (98%) participants. IHC was the most wide-spread method for ALK testing and was stated to be established in 50 institutions (89%), followed by ISH (46, 82%) and NGS (33, 59%). For routine diagnostics, IHC and ISH was used by 38 (68%) and 31 (55%) participants, only 15 (27%) used NGS. 45 (79%) participants also described if they relied on one method or if they combined different techniques. IHC was used as the only method by 23 institutions (51%). Seven (16%) and three (7%) participants solely relied on ISH or NGS, respectively. Eight (18%) and four (9%) institutions used IHC in combination with ISH or NGS for all cases, respectively.

#### IHC

Out of 38 participating institutions, 33 (87%) successfully passed the ring trial using IHC. The median score was 19.3 points. Twenty-nine participants (77%) reached the full score of 20 points. Nineteen points were achieved by one (3%), 18 points by three (8%), 17 points by one (3%), and 16 points by four institutions (11%).

In total, 380 individual IHC-based evaluations were reported. Three hundred sixty-six (96%) were correct, 12 (3%) were incorrect, and two (0.5%) were not evaluable due to technical issues. The sensitivity was 92% while the specificity was 100%. Regarding the interrater reliability, the Fleiss’ kappa value was 0.888.

**Fig. 3:**
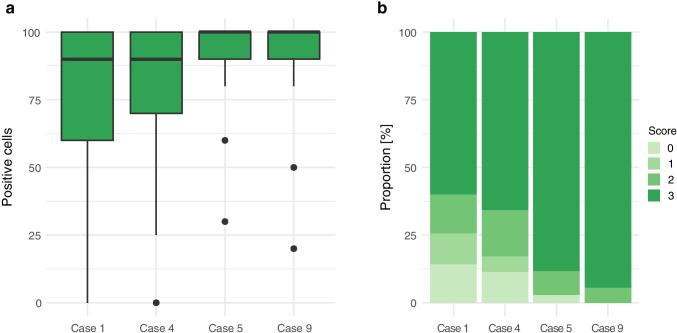
Results of the immunohistochemical evaluation**. a** Boxplot showing the distribution of positive cells for the four ALK positive cases. **b** Stacked bar plots summarizing the proportion of the different scores that were assessed in the four ALK positive specimens

The median proportion of positive tumor cells was at least 90% for all four ALK positive cases (Fig. 3a). Furthermore, the vast majority of participants observed a strong staining pattern (= Score 3), especially in Cases 5 and 9 (Fig. 3b).

The most commonly used antibody clone was D5F3 (17 participants, 45%), followed by 1A4 (15 participants, 40%), 5A4 (five participants, 13%), and ALK1 (one participant, 3%).

Detailed information regarding the antibodies, their respective manufacturers, the dilutions, as well as the sensitivities for each clone and antibody are summarized in Table 1.

**Table 1:** Detailed information on the different antibodies that have been used in the ring trial as well as their respective sensitivities

Manufacturer	Clone	Dilution	Participants	Successful (%)	Antibody sensitivity	Clone sensitivity
Leica	5A4	1:20—1:50	2	2 (100%)	100%	95%
Novocastra	5A4	1:25—1:100	2	2 (100%)	100%	
Zytomed	5A4	1:50	1	1 (100%)	90%	
Origene	1A4	1:100—1:250	5	5 (100%)	100%	95%
Zeta	1A4	1:40	1	1 (100%)	100%	
Zytomed	1A4	1:50 to 1:400	9	8 (89%)	92%	
Ventana	ALK1	Ready to use	1	1 (100%)	100%	100%
Cell Signaling	D5F3	1:50—1:1000	8	6 (75%)	92%	95%
Ventana	D5F3	Ready to use	9	7 (78%)	89%	

Following the external ring trial, the stained IHC slides of the five institutions that did not pass the ring trial were re-evaluated centrally at the Institute of Pathology of the Charité–University Hospital Berlin. In two cases, the specimens that were falsely classified as negative did not show any immunoreactivity. For the remaining three cases, we observed weak or aberrant (stippled) staining patterns that were incorrectly considered as ALK negative (Supplementary Figure S1).

#### ISH

Out of 29 participants (97%), 28 successfully participated in the ring trial using ISH. The mean score was 19.5 points.

Out of a total of 290 individual ISH-based analyses, 281 (97%) were correct, six (2%) were incorrect, and three tests (1%) failed due to technical issues. The sensitivity was 94% (102/108) while the specificity was 100% (171/171). The Fleiss’ kappa value was 0.896.

**Fig. 4:**
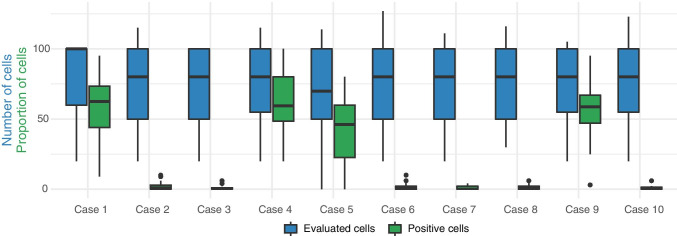
Box plots showing the results from in-situ hybridization. The distribution of evaluated cells for each case is shown in blue while the distribution of positive cells for each case is shown in green

The number of evaluated cells, as well as the number of cells with signals consistent with translocation, are summarized in Fig. 4. There was no significant difference in the number of evaluated cells between different cases. For each case, between 20 and 127 cells were analyzed with a mean of 76 cells. The median proportion of cells with non-fusion signals was 59% for Case 1, 63% for Case 4, 42% for Case 5 and 56% for Case 9.

FISH was used by 22 (76%) institutions. Chromogenic in situ hybridization (CISH) was used by seven participants (24%). The participant that did not successfully pass the ring trial used CISH.

The Zytovision SPEC ALK DualColor Break Apart Probe was used by 17 institutions (59%), followed by the Zytovision SPEC ALK/EML4 Tri Check Probe (seven participants, 24%), the Abbot Molecular Vysis LSI ALK Dual Color Probe (five institutions, 17%), and the Zytovision FlexISH® ALK/ROS1 DistinguISH Probe as well as the Cytocell ALK Breakapart Probe (each one participant, 3%; Table 2).

**Table 2:** Detailed information on the different in-situ hybridization probes that have been used in the ring trial

Manufacturer	Probe	Participants	Successfull (%)
Zytovision	SPEC ALK DualColor Break Apart Probe	15	14 (93%)
Zytovision	SPEC ALK/EML4 Tri Check Probe	7	7 (100%)
Abbott Molecular	Vysis LSI ALK Dual Color	5	5 (100%)
Zytovision	FlexISH® ALK/ROS1 DistinguISH Probe	1	1 (100%)
Cytocell	ALK Breakapart Probe	1	1 (100%)

### RNA/DNA sequencing

Overall, 18 of 19 participants (95%) successfully passed the ring trial using RNA/DNA sequencing methods. The mean score was 19.5 points.

Fifteen institutions (79%) reached the maximum score of 20. Two participants (11%) scored 19 points as they each classified one case as “not suitable” due to technical reasons. One institution (5%) achieved 18 points and did not detect the presence of an ALK inversion in one case (Case 9). Furthermore, one participant (5%) reached eight points, as six cases were reported “not suitable” due to technical reasons.

Out of a total of 190 individual NGS-based analyses, 181 (95%) were correct, one (0.5%) was incorrect, and eight tests (4%) failed due to technical issues. The sensitivity was 98.6% while the specificity was 100%. The Fleiss’ kappa value was 0.975.

Data on the respective assay was provided by 16 institutions. The most commonly used assay was Oncomine Focus (Thermo Fisher Scientific; five participants, 31%), followed by FusionPlex Lung (Archer; four institutions, 25%), NEOselect onsite (NEO New Oncology; two participants, 13%), AmpliSeq for Illumina Focus Panel (Illumina; two participants, 13%), and TruSight Tumor 170 (Illumina; one participant, 6%).

Data on the detected ALK variant was submitted by ten participants, with concomitant results in all cases. Cases 1, 4, and 5 harbored the variant V1, while the variant V3a was present in Case 9. Additionally, the amount of RNA/DNA input was specified by 15 institutions. The detailed data is summarized in Supplementary Table S1. Of note, the only false-negative report also had the lowest nucleic acid input of all cases within the whole EQA scheme (8 ng).

## Discussion

FISH is still regarded as the gold standard and diagnostic method of choice to detect ALK-positive NSCLC [3, 4]. However, in the last years, further methods such as IHC and NGS showed promising and even comparable results and have been integrated in the daily routine testing [16, 17]. EQA schemes may serve to show the status quo of the diagnostic standard (quality) in a multi-center setting. To this end, based on an initial so-called internal ring trial to choose and validate eligible tumor samples, we enrolled an external nationwide ring trial encompassing 57 participants. Thus, we were for the first time able to evaluate the interrater concordance of IHC, ISH, and RNA/DNA sequencing to reliably identify ALK alterations in NSCLC between different laboratories of pathology.

In line with previous reports, we observed a high sensitivity and interrater reliability for ISH [6, 18, 19]. Of note, only clearly positive cases were included in the ring trial. Therefore, the reported sensitivity for ISH does not account for so-called borderline cases with translocation signals near the cut-off or the rare but existing IHC negative but FISH positive cases [24, 25].

Implementation of diagnostic ALK IHC was initially complicated by the existence of a whole variety of different antibody clones as well as the lack of standardized protocols and scoring systems [19]. However, after several successful harmonization studies, IHC quickly became a reliable screening method [5, 6]. In our study, we observed an adequate sensitivity and interrater agreement demonstrating the reliability of IHC across different institutions. In comparison to ISH and NGS, these values (and also the number of institutions with successful participation) were relatively low. However, a central reevaluation of the IHC slides from the institutions that did not successfully participate in the ring trial revealed that false negative results were primarily due to misinterpretation and not only due to technical issues. In more than half of the re-evaluated false negative cases, we observed a weak or an aberrant (stipple staining) staining pattern. For these specimens, a second method should be used to determine if an ALK translocation is present or not [26, 27].

In line with current recommendations, the most commonly used antibody clone was D5F3 [28]. The D5F3 antibody by Ventana is also approved by the FDA for selection of patients to be treated with Crizotinib. In comparison to other investigations, the sensitivity of this clone in our study was lower, but still within the range of the values reported in literature [6, 18, 19, 27]. The second most commonly used antibody was 1A4. This clone has not yet been validated in a multicenter setting before but showed promising results in previous reports [29]. In line with these studies, we observed a higher sensitivity compared with D5F3, further supporting the suitability of 1A4 for diagnostic use.

Regarding RNA/DNA sequencing, this is the first study to investigate this method in a multicenter setting. Interestingly, the observed sensitivity and interrater agreement was higher than for IHC and ISH. In fact, only one false negative result was observed, which was most likely caused by low RNA input. Compared with IHC and ISH, the number of samples that were not evaluable was considerably larger, although this was mainly caused by one participant who was unable to extract a sufficient amount of RNA/DNA in six of ten cases. It is well known that formalin fixation causes molecular modification, fragmentation and degradation of nucleic acids [30]. However, as no other institution observed compromised RNA/DNA quality, this outlier could be caused by individual technical difficulties during the extraction process.

The use of NGS panels (DNA and/or RNA fusion panels) comes with multiple benefits. Most obviously, these panels usually cover other predictive and prognostic molecular alterations in multiple genes, such as EGFR, ROS1, RET or MET. Furthermore, in contrast to ISH and IHC, RNA/DNA sequencing can be used to determine the ALK fusion variant, which, in future, could be a relevant information for refined treatment decisions [13]. Despite these advantages, the use of RNA/DNA sequencing is also limited, mainly by the required technical expertise, relatively high cost and longer turn-around time as well as potentially by RNA/DNA quality. Therefore, ISH and IHC will probably still be required in the future.

In addition to the main results from the ring trial, we also gained detailed insight on the distribution and application of the different techniques for ALK testing. ALK IHC is a relatively cheap and reliable method, which is established in almost all institutions and is also most commonly used for ALK testing. In fact, more than half of the participating institutions exclusively used IHC in routine diagnostics. Our investigation also showed that over 50% of the participating institutions have already established RNA/DNA sequencing for the detection of ALK fusions. However, only half of them actually used this method in the routine diagnostic setting. The excellent results for RNA/DNA sequencing that were observed in the multicentric validation presented in this study further encourages the broad implementation and application of this technique in the routine diagnostic of ALK translocation in NSCLC, although this approach can be limited by the small size of most biopsy specimens.

A limitation of our study is the relatively small number of samples that have been used for this EQA scheme. Although the number is comparable with previous studies [6, 10], a larger set of cases might be helpful to improve the robustness of the obtained results.

## Conclusion

In summary, we provide further proof that the ISH- and IHC-based identification of ALK translocations in NSCLC is highly reliable and reproducible between different pathology laboratories. Furthermore, we show that RNA/DNA sequencing might even be superior to IHC and ISH in terms of specificity and interrater reliability. However, the application of this method in routine diagnostics might be limited by relatively high cost, required technical expertise, and tissue quality.

## Supplementary Information

Below is the link to the electronic supplementary material.

Supplementary file1 (PDF 197 KB)
