# Supplementary material for: Status quo of ALK testing in lung cancer: results of an EQA scheme based on in-situ hybridization, immunohistochemistry, and RNA/DNA sequencing
Source: Virchows Arch. 2021 Jun 25;479(2):247–55. doi: 10.1007/s00428-021-03106-5 (PMC8364534; doi:10.1007/s00428-021-03106-5)
Supplement: Supplementary file 1 — Supplementary file1 (PDF 197 KB) [file 428_2021_3106_MOESM1_ESM.pdf]

Participant	Case 1	Case 2	Case 3	Case 4	Case 5	Case 6	Case 7	Case 8	Case 9	Case 10
1	15	15	15	15	15	15	15	15	15	15
2	250	250	250	250	250	250	250	250	250	250
3	200	200	144	97	200	200	110	48	200	194
4	154	150	150	161	84	164	165	133	98	147
5	31	34	60	24	21	44	20	29	16	50
6	15	20	20	15	15	15	20	20	15	15
7	50	50	50	50	50	50	50	50	50	50
8	50	50	100	50	100	100	100	100	100	100
9	85	76	85	65	42	85	42	51	8	85
10	99	189	219	130	83	265	94	74	62	232
11	150	150	150	150	150	150	150	150	150	150
12	200	200	158	200	200	200	200	200	200	200
13	100	100	100	100	100	100	100	100	100	100
14	10	10	10	10	10	10	10	10	10	10
15	250	250	250	250	216	250	249	180	250	250
Median	99	100	100	97	84	100	100	74	98	100
Minimum	10	10	10	10	10	10	10	10	8	10
Maximum	250	250	250	250	250	265	250	250	250	250

Supplementary Table S1 Table summarizing the amount of nucleic acids for RNA/DNA sequencing in nanograms.

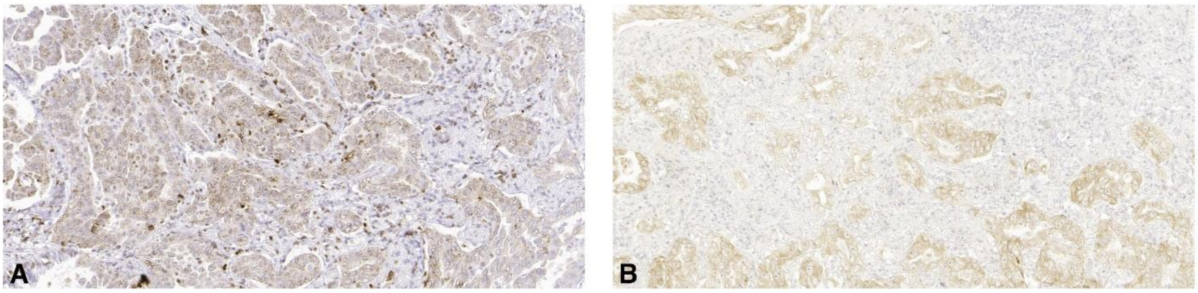

Supplementary Figure S1 Staining patterns that were observed during the central reevaluation of immunohistochemical (IHC) stains that were falsely classified as ALK negative.

A Representative image of a case with weak immunoreactivity and an aberrant, so-called “stippled staining” pattern.

B Representative image of an IHC staining with weak positivity.
